# Diagnosis of shoulder apprehensions two years after latarjet procedure: a retrospective observational study

**DOI:** 10.1080/07853890.2025.2557506

**Published:** 2025-09-13

**Authors:** Elio Disegni, Marina Torrens, Geoffroy Nourissat

**Affiliations:** Clinique de l’épaule, Clinique des Maussins, Ramsay Santé, Paris, France

**Keywords:** Shoulder instability, glenohumeral dislocation, latarjet procedure, capsular repair, shoulder apprehension

## Abstract

**Introduction:**

Despite high stabilization rates after Latarjet procedure, persistent apprehension during shoulder use remains a concern.

**Methods:**

Retrospective observational single-center study in patients who underwent Latarjet procedure for shoulder dislocation. The primary objective was two-year post-operative overall apprehension rate (‘In general, do you experience any apprehension when using your shoulder?’), with or without capsular repair. The secondary objective was to assess whether overall apprehension was related to functional scores (Rowe, Walch-Duplay and SANE).

**Results:**

Fifty-three patients were screened (mean age, 30.1 years; capsular repair, 83.0%) and 44 were analyzed for primary and secondary objectives. No patient experienced shoulder dislocation recurrence. Overall apprehension for using shoulder was nevertheless high and comparable with or without capsular repair (57.1% vs. 50.0%, respectively; *p* = 1.00). According to Stability domains of Walch-Duplay and Rowe scores, 20.5% and 29.5% of patients had shoulder apprehension, respectively. Specificity of Rowe, Walch-Duplay and SANE scores to identify overall apprehension was 94.4% (95% CI, 83.9–100), 94.7% (84.7–100), 77.8% (58.6–97.0) and sensitivity was 47.8% (95% CI, 27.4–68.2), 47.8% (27.4–68.2), 72.7% (54.1–91.3), respectively.

**Conclusion:**

Half of patients reported persistent shoulder apprehension after Latarjet procedure regardless of capsular repair. Rowe and Walch-Duplay scores had high specificity to identify shoulder apprehension. However, their low sensitivity may underestimate persistent shoulder apprehension despite obvious shoulder stability.

## KEY MESSAGES

Despite high stabilization rates following the Latarjet procedure, nearly half of patients still experience persistent apprehension when using their shoulder.

Traditional functional scores like Rowe and Walch-Duplay may not fully capture this issue.

Rehabilitation efforts should focus on improving confidence and functional use of the shoulder, as some patients may still feel apprehensive even without dislocation recurrence.

## Introduction

The shoulder joint is particularly exposed to the risk of instability, which can manifest as recurrent dislocations or painful subluxations. Shoulder dislocation is common in athletes and most often occurs in the anterior direction [1]. During dislocation, the head of the humerus leaves the glenoid cavity and passes in front of the scapula. During movement, the bone may be damaged, resulting in small fractures of humerus, glenoid, or both. In cases of bone lesions, the risk of recurrence is high, particularly when the patient is young and sporty [2]. In this situation, the Latarjet procedure should be proposed, which consists in transferring the coracoid process and the attached tendon to the anterior glenoid margin [2]. Depending on the surgeon’s experience, this operation can be performed by conventional minimally invasive technique or more recently by arthroscopy [3].

Despite a high stabilization rate after Latarjet procedure [4], the main problem reported by patients is apprehension about using their shoulder [5]. Apprehension has been defined as anxiety and motor resistance after episodes of glenohumeral dislocation [6]. Even if the shoulder is clinically stable and patients return to sport, patient apprehension may persist after surgery in 3%–51% of cases, depending on the studies [6]. Physiopathology of shoulder apprehension remains unclear and various hypotheses have been proposed [6]. Interestingly, functional magnetic resonance imaging studies suggest that repeated dislocations changed neural connectivity in brain areas specialized in the cognitive control of motor behavior [7–10].

The capsule is a critical structure that contributes to the overall stability of the joint and capsular repair during Latarjet procedure is thought to be an additional stabilizing mechanism [11]. Capsular repair is also thought to improve proprioception, thus helping to reduce patient apprehension.

In this study, we hypothesized that capsular repair added to the Latarjet procedure decreased postoperative patient apprehension to use their shoulder. Another objective was to assess whether apprehension was adequately appraised by functional scores.

## Materials and methods

### Study design and eligibility criteria

This was a retrospective, observational, single-center study in patients who underwent a Latarjet procedure in our center. The primary objective was to compare the rates of overall apprehension reported by patients two years after Latarjet procedure with or without capsular repair. The secondary objective was to assess whether overall apprehension was related to the functional scores (Walch-Duplay Rowe and SANE).

Patients were included consecutively if they were over 18 years of age, had undergone an open Latarjet procedure and had a 2-year post-operative follow-up.

The study was conducted in accordance with the Declaration of Helsinki. This study was approved by the Ethics Committee/IRB (IRB00010835) of the Scientific Committee of Ramsay Santé for Education and Research (COS-RGDS-2024-02-003-NOURISSAT-G; February 20, 2024). This Ethics Committee/IRB waived patient consent. Nevertheless, patients were informed in writing about the re-use of their health data, and have been given the opportunity to object in writing to their participation. The study complied also with the French regulatory methodology MR-004 for health-related studies. MR-004 is a methodology adopted by the French Data Protection Authority for all studies that re-use health data already collected for care or research purposes (e.g. retrospective observational studies).

### Surgical procedure

The Latarjet technique was performed *via* a deltopectoral approach with the patient in the supine position. The coracoacromial ligament was sectioned laterally, and the pectoralis major was was sectioned along the medial edge of the coracoid. The coracoid was osteotomized at its knee. Two holes were drilled in the graft: a central 2.5 mm hole 5 mm from the lower edge and an 11-mm upper hole, slightly medial. The graft was fixed to the glenoid using two screws through a horizontal split of the subscapularis at the junction of its upper two-thirds and lower one-third. The capsule was incised in an inverted T shape. A first anchor and 3.5-mm screw were placed in the lower glenoid. A second screw was inserted through the upper hole of the graft into a new glenoid hole. A second anchor was placed at the anterior glenoid, at the top of the graft [12]. The capsule was repaired using the anchors’ threads, except in patients from the ‘without capsular repair’ group.

### Data collected

Study data collected from consecutive medical records of eligible patients were age at surgery, laterality, number of dislocations, sport practice, anterior apprehension test and Gagey’s hyperabduction test before surgery, time between first dislocation and surgery, bone lesions and localizations, capsular repair or not, number of anchors, overall apprehension, Walch-Duplay, Rowe and single alpha-numeric evaluation (SANE) scores two years after surgery.

Overall apprehension was assessed by asking the patient: ‘In general, do you experience any apprehension when using your shoulder? (yes/no)’.

The Walch-Duplay score is a 100-point functional score assessing shoulder stability according to four 25-point criteria: sport, stability, mobility and pain [13]; it is categorized in 4 classes: poor (≤ 50), medium (51–75), good (76–90) and excellent (≥ 91).

The Rowe score is a 100-point functional score based on 4 items: function (50 points), stability (30 points), mobility (10 points) and pain (10 points) [14]; it is categorized in 4 classes: poor (≤ 50), medium (51–74), good (75–89) and excellent (≥ 90).

The SANE score assesses patient self-reported shoulder function by asking ‘How would you rate your shoulder today as a percentage of normal (0% to 100% scale with 100% being normal)?’ [15].

### Statistical analysis

The primary endpoint was the rate of patients with an overall apprehension about using their shoulder two years after Latarjet procedure with or without capsular repair. Shoulder apprehension was also assessed with the scores of the Stability domain of the Walch-Duplay and Rowe scores.

To assess whether overall apprehension was related to functional scores, the specificity and sensitivity of each functional score to discriminate between patients with or without global apprehension were assessed. A ROC analysis was performed and the area under the curve (AUC) was calculated for each functional score. The thresholds for the Rowe, Walch-Duplay and SANE scores for the best estimate of overall apprehension were determined using the Youden index and sensitivity and specificity were calculated for each functional score.

Categorical variables were compared using Chi-square test or Fisher’s exact test. Quantitative variables were compared using Student’s t-test or Wilcoxon test. All comparisons were made at a statistical significance level set at *p* < 0.05.

Analyses were performed using SAS software, version 9.4 (SAS Institute Inc., Cary, NC, USA).

## Results

### Patients disposition and characteristics

**Figure 1.**
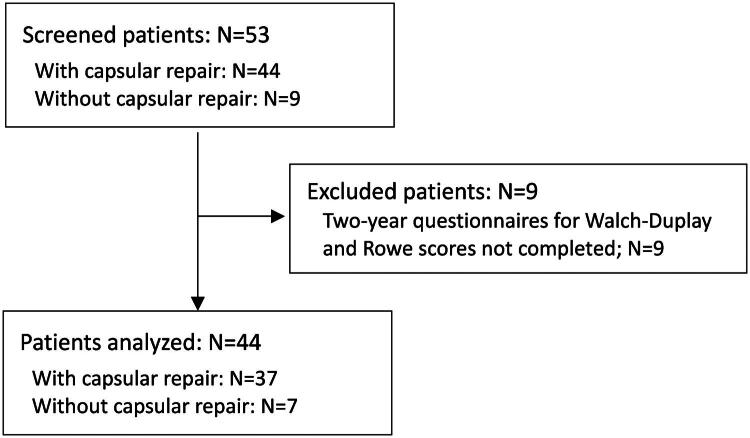
Flow chart.

A total of 53 patients who underwent surgery between January and December 2021 were screened (44 with capsular repair and 9 with no capsular repair). Nine patients did not complete the questionnaire (Walch-Duplay and Rowe scores) and were excluded from the analysis population (*n* = 44; 37 with capsular repair and 7 with no capsular repair) (Figure 1). The rate of patients with capsular repair was comparable in analyzed patients vs. excluded patients (84.1% vs. 77.8%; *p* = 0.649). Other characteristics were comparable, except for mean time between first dislocation and surgery (55.6 ± 71.5 months in analyzed patients vs. 19.7 ± 15.3 in excluded patients; *p* = 0.0276); this difference was however not significant by classes (*p* = 0.1245).

Mean age at the time of surgery was 27.7 years in patients with capsular repair and 41.5 years in patients with no capsular repair (*p* = 0.0083) (Table 1). A large number of patients practiced sport (85.1%). A total of 43.1% of patients had experienced ≥ 5 dislocations prior to surgery. Almost all patient had a positive anterior apprehension test (91.7%) and positive Gagey’s hyperabduction test (89.6%) before surgery. Bone lesions on humerus were frequent (65.0% on humerus only and 22.5% on both humerus and glenoid) (Table 1).

**Table 1.** Patient characteristics (screened population).

	Capsular repair (*N* = 44)	No capsular repair (*N* = 9)	Analysis population (*N* = 53)
Age at surgery, years			
Mean (SD)	27.7 (8.1)	41.5 (14.2)^a^	30.1 (10.6)
< 20	30 (68.2)	3 (33.3)	33 (62.3)
Laterality, *n* (%)			
Right	22 (50)	5 (55.6)	27 (50.9)
Left	22 (50)	4 (44.4)	26 (49.1)
Number of dislocations, *n* (%)			
< 5	25 (59.5)	4 (44.4)	29 (56.9)
≥ 5	17 (40.5)	5 (55.6)	22 (43.1)
Missing	2	0	2
Sport, *n* (%)	33 (84.6)	7 (87.5)	40 (85.1)
Missing	5	1	6
Anterior apprehension test, *n* (%)	37 (90.2)	7 (100)	44 (91.7)
Missing	3	2	5
Gagey’s hyperabduction test, *n* (%)	36 (87.8)	7 (100)	43 (89.6)
Missing	3	2	5
Time between first dislocation and surgery			
Mean (SD), months	37.7 (40.8)	97.4 (120.6)	48.9 (66.2)
Classes, *n* (%)			
< 1 year	8 (20.5)	1 (11.1)	9 (18.8)
[1–4] years	18 (46.2)	2 (22.2)	20 (41.7)
≥ 4 years	13 (33.3)	6 (66.7)	19 (39.6)
Missing	5	0	5
Bone lesions, *n* (%)			
Humerus only	24 (68.6)	2 (40.0)	26 (65.0)
Humerus and glenoid	7 (20.0)	2 (40.0)	9 (22.5)
Glenoid only	4 (11.4)	1 (20.0)	5 (12.5)
Missing	9	4	13
Number of anchors, *n* (%)			
0	0	9 (100)	9 (17.0)
1	2 (4.5)	0	2 (3.8)
2	42 (95.5)	0	42 (79.2)

^a^*p* = 0.0083 for capsular repair vs. no capsular repair (Wilcoxon test).

Two years after the surgery, mean (SD) SANE score was 63.0 (38.0) (one missing data). A total of 61.4% of patients had returned to sport at the same level and 11.4% at a lower level (Table 2); 13.6% had changed of sport and 13.6% had stopped sport. During the 2-year follow-up, no recurrence of dislocation was reported (3 missing data).

**Table 2.** Watch duplay score two years after Latarjet procedure.

	Capsular repair (*N* = 37)	No capsular repair (*N* = 7)	Analysis population (*N* = 44)	*p*-value
Sports activity or daily routine if no sport practiced,				
Mean (SD)	18.7 (8.9)	16.4 (11.8)	18.4 (9.3)	0.6863^a^
Return to the same sport at the same level/No discomfort	23 (62.2)	4 (57.1)	27 (61.4)	0.5016^b^
Return to the same sport but at a lower level/Slight discomfort during vigorous movements	4 (10.8)	1 (14.3)	5 (11.4)	
Change of sport/Slight discomfort during simple movements	6 (16.2)	0	6 (13.6)	
Stopping sport/Severe discomfort	4 (10.8)	2 (28.6)	6 (13.6)	
Stability				
Mean (SD)	21.5 (6.7)	23.6 (3.8)	21.8 (6.3)	0.4699^a^
No apprehension	27 (73.0)	6 (85.7)	33 (75.0)	1.0000^b^
Persistent apprehension	8 (21.6)	1 (14.3)	9 (20.5)	
Feeling of instability	2 (5.4)	0	2 (4.5)	
Pain	19.7 (7.5)	18.6 (9.4)	19.5 (7.8)	0.8397^a^
Mobility	21.0 (6.7)	19.3 (9.3)	20.8 (7.0)	0.6808^a^
Total score	81.0 (21.9)	77.9 (27.1)	80.5 (22.5)	0.8954^a^
Walch-Duplay score classes, n (%)				
Poor	3 (8.1)	1 (14.3)	4 (9.1)	0.8836^b^
Medium	10 (27.0)	1 (14.3)	10 (25.0)	
Good	13 (35.1)	3 (42.9)	16 (36.4)	
Excellent	11 (29.7)	2 (28.6)	13 (29.5)	

^b^Fisher’s exact test; ^a^Wilcoxon test.

### Shoulder apprehension assessments two years after surgery

According to the Stability domain of the Walch-Duplay score, 20.5% of patients had persistent apprehension two years after surgery (Table 2); according to the Stability domain of the Rowe score, 29.5% of patients were still apprehensive about placing the arm in certain positions (Table 3). For both scores, apprehension rates were comparable with or without capsule repair.

**Table 3.** Rowe score two years after Latarjet procedure.

	Capsular repair (*N* = 37)	No capsular repair (*N* = 7)	Analysis population (*N* = 44)	*p*-value
Function, mean (SD)	42.6 (11.5)	35.7 (24.4)	41.5 (14.1)	0.9850^a^
Pain, mean (SD)	8.2 (2.7)	6.4 (3.8)	8.0 (2.9)	0.1765^a^
Stability,				
Mean (SD)	23.5 (8.8)	23.6 (8.0)	23.5 (8.6)	1.0000^a^
No recurrence, subluxation or apprehension, n (%)	23 (62.2)	4 (57.1)	27 (61.4)	
Apprehension about placing the arm in certain positions, n (%)	10 (27.0)	3 (42.9)	13 (29.5)	
Subluxation (not requiring reduction), n (%)	3 (8.1)	0	3 (6.8)	
Positive apprehension test or notion of instability, n (%)	1 (2.7)	0	1 (2.3)	
Mobility, mean (SD)	6.8 (3.2)	7.9 (2.7)	6.9 (3.1)	0.4292^a^
Total Rowe score	81.1 (18.5)	73.6 (31.2)	79.9 (20.7)	1.0000^a^
Rowe score classes, n (%)				
Poor	3 (8.1)	2 (28.6)	5 (11.4)	0.4099^b^
Medium	4 (10.8)	0	4 (9.1)	
Good	16 (43.2)	2 (28.6)	18 (40.9)	
Excellent	14 (37.8)	3 (42.9)	17 (38.6)	

^a^Wilcoxon test.

^b^Fisher’s exact test.

Overall apprehension for using shoulder was reported in 56.1% (23/41; 3 missing data). This rate was comparable in patients with or without capsular repair: 57.1% (20/35) vs. 50.0% (3/6), respectively (*p* = 1.00).

### Specificity and sensitivity of functional scores to identify overall apprehension

Mean Walch-Duplay scores were significantly lower in patients with overall apprehension compared to patients without overall apprehension: 19.8 vs. 23.9 (*p* = 0.0432) for stability score and 70.1 vs. 93.3 for total score (*p* = 0.0003), respectively (Table 4). Similarly, mean Rowe scores were significantly lower in patients who reported overall apprehension after 2 years compared to patients without overall apprehension: 19.1 vs. 28.1 (*p* = 0.0013) for stability score and 70.4 vs. 93.1 for total score (*p* < 0.0001), respectively (Table 4). The mean (SD) SANE score was 71.5 (25.1) in patients with overall apprehension and 90.3 (9.3) in those without overall apprehension (*p* = 0.0003).

**Table 4.** Walch-Duplay and Rowe scores according to overall apprehension.

	Overall apprehension (*N* = 23)	No overall apprehension (*N* = 18)	*p*-value
Walch-Duplay Stability score			
Mean (SD)	19.8 (7.8)	23.9 (3.2)	0.0432^a^
No apprehension, *n* (%)	14 (60.9)	16 (88.9)	
Persistent apprehension, *n* (%)	7 (30.4)	2 (11.1)	
Feeling of instability, *n* (%)	2 (8.7)	0	
Total score	70.1 (25.4)	93.3 (8.6)	0.0003^a^
Walch-Duplay score classes, *n* (%)			
Poor	4 (17.4)	0	0.0010^b^
Medium	9 (39.1)	1 (5.6)	
Good	8 (34.8)	7 (38.9)	
Excellent	2 (8.7)	10 (55.6)	
Rowe Stability score			
Mean (SD)	19.1 (8.7)	28.1 (5.7)	0.0013^a^
No recurrence, subluxation or apprehension, n (%)	8 (34.8)	16 (88.9)	
Apprehension about placing the arm in certain positions, n (%)	12 (52.2)	1 (5.6)	
Subluxation (not requiring reduction), n (%)	2 (8.7)	1 (5.6)	
Positive apprehension test or notion of instability, n (%)	1 (4.3)	0	
Total Rowe score, mean (SD)	70.4 (21.4)	93.1 (8.4)	<0.0001^a^
Rowe score classes, n (%)			
Poor	4 (17.4)	0	0.0003^b^
Medium	4 (17.4)	0	
Good	12 (52.2)	5 (27.8)	
Excellent	3 (13.0)	13 (72.2)	

^a^Wilcoxon test.

^b^Fisher’s exact test.

**Figure 2.**
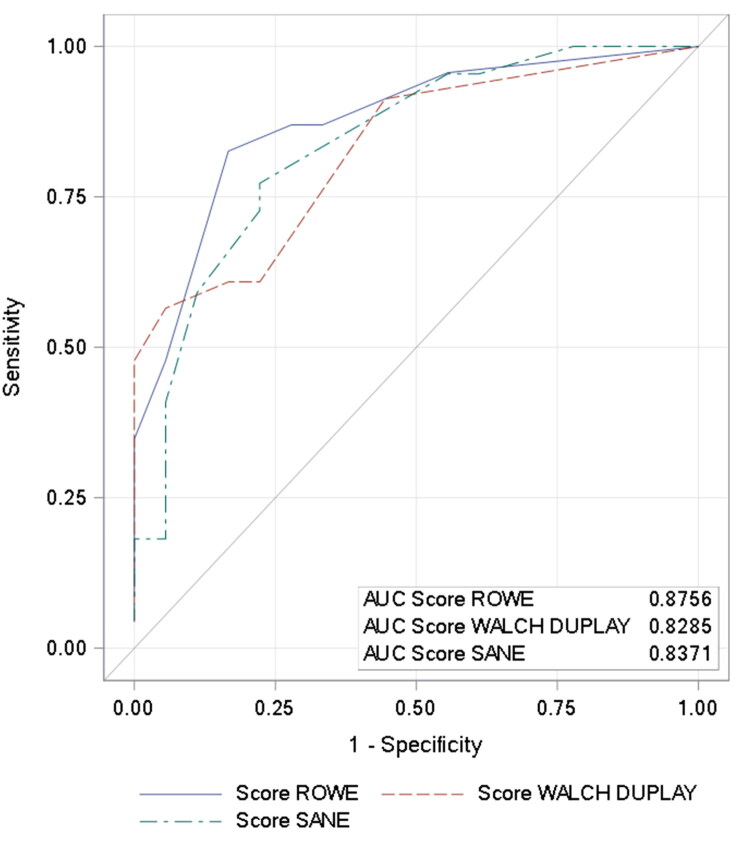
ROC curves of Rowe, Walch-Duplay and SANE scores for discriminating between patients with or without overall apprehension.

A ROC analysis was performed in order to define sensitivity and specificity of the Rowe, Walch-Duplay and SANE scores for discriminating between patients with or without overall apprehension. The area under curve was 0.88 (95% CI, 0.77–0.98) for Rowe score, 0.83 (95% CI, 0.71–0.95) for Walch-Duplay and 0.84 (95% CI, 0.71–0.96) for SANE score. All AUCs were >0.8 and therefore had satisfactory discriminating power to detect global apprehension. The calculated optimal cut-offs of functional scores were 80, 75 and 87, respectively (patients with a score below the cut-off were considered to have overall apprehension in using their shoulder). With these cut-offs, specificity of Rowe, Walch-Duplay and SANE scores to identify overall apprehension was 94.4%, 94.7% and 77.8%. The respective values for sensitivity were 47.8%, 47.8% and 72.7% (Table 5 and Figure 2).

**Table 5.** Specificity and sensitivity of Rowe, Walch-Duplay and SANE scores to identify overall apprehension.

	Overall apprehension (*N* = 23)	No overall apprehension (*N* = 18)	Specificity Sensitivity	95% CI
Rowe score, *n* (%)				
≥ 80	12 (52.2)	17 (94.4)	Sp: 94.4%	83.9–100
< 80	11 (47.8)	1 (5.6)	Se: 47.8%	27.4–68.2
Missing	0	0		
Walch-Duplay score, *n* (%)				
≥ 77	12 (52.2)	18 (100)	Sp: 94.7%	84.7–100
< 77	11 (47.8)	0	Se: 47.8%	27.4–68.2
Missing	0	0		
SANE score, *n* (%)				
≥ 87	6 (27.3)	14 (77.8)	Sp: 77.8%	58.6–97.0
< 87	16 (72.7)	4 (22.2)	Se: 72.7%	54.1–91.3
Missing	1	0		

Note: Patients with a score below the cut-off are considered to have overall apprehension in using their shoulder.

Abbreviations: Se, sensitivity; Sp, specificity.

### Predictive factors of Walch-Duplay and Rowe scores

The Walch-Duplay score was rated good or excellent in 65.9% of patients (Table 2) and the Rowe score was rated good or excellent in 79.5% of patients (Table 3). No multivariate model could be built to define predictive factors of Walch-Duplay or Rowe scores (poor or medium vs. good or excellent) using the following factors: capsular repair, age, bone lesions, number of dislocations, time between first dislocation and surgery.

## Discussion

In our cohort, half of patients reported persistent shoulder apprehension two years after Latarjet procedure. Statistical significance was not achieved for the comparison of apprehension rates after procedures with or without capsular repair. In addition, the small number of patients who did not undergo capsular repair and the heterogeneity of the two groups prevent drawing definitive conclusions on this issue. Therefore, our initial hypothesis that capsular repair could improve shoulder apprehension remains unanswered.

The capsular repair reinforces the soft tissue constraints and is thought to concur to the stabilizing effect of the Latarjet procedure. However, clinical studies that compared functional outcomes after Latarjet procedure in patients with or without capsular repair are scarce. The study of Sahu compared the external rotation range and clinical outcomes in 30 patients who underwent Latarjet procedure with coracoacromial capsule repair (prospective arm) and 30 patients without any capsular repair (retrospective arm) [16]. After a follow-up of at least one year of each patient, capsular repair led to no or minimal limitation of external rotation. In addition, Walch-Duplay score, Rowe score and Subjective Shoulder Value were comparable in the two groups. The study of Kim et al. in 47 patients undergoing Latarjet procedure (23 with capsular repair and 22 without) reported that the external rotation deficit at one year after surgery and all other clinical scores were comparable in the two groups with or without capsular repair [17]. These studies, which did not support the capsular repair as an essential step in Latarjet procedure, are consistent with our results. Nevertheless, taken all together, these results underline the need for large-scale and, if possible, randomized studies to definitively conclude on the utility of adding capsular repair to the Latarjet procedure.

Of interest, the functional scores (Walch-Duplay, Rowe and SANE) at two years after surgery of our cohort were significantly lower in patients who reported shoulder apprehension. The relationship between shoulder function and apprehension was further explored by ROC analysis in order to define the sensitivity and the specificity of the functional scores in discriminating patients with or without overall apprehension. Functional scores, specifically Rowe and Walch-Duplay score, had a high specificity. In other words, there was a close relationship between improved shoulder function (score above cut-off) and the absence of overall apprehension. The results for sensitivity were less satisfactory, meaning that a large number of patients had correct functional scores, but nevertheless complained of overall apprehension.

Pre- and postoperative shoulder apprehension appears to be more complex than a simple mechanical shoulder disorder and its physiopathology remains unclear [6]. Patients with shoulder apprehension after surgery avoid any shoulder movement despite a clinically stable joint. It has been hypothesized that repeated dislocations induced brain changes [7–10]. Thus, using functional magnetic resonance imaging in patients suffering from shoulder apprehension, Haller et al. showed that neural connectivity was modified in several cerebral areas involved in the cognitive control of motor behavior [8]. Other hypotheses rely on peripheral neuromuscular lesions or on persisting mechanical instability caused by micro-movements [6]. At present, there is no clinical score that could account for the cognitive changes induced by apprehension. Being aware that shoulder apprehension may be the consequence of brain changes could help avoid costly, unnecessary and potentially dangerous investigations. A promising therapeutic approach would be not to focus exclusively on the glenohumeral joint, but to consider more adapted treatments such as biofeedback or cognitive-behavioral therapy [6]. Thus, in patients with functional tests in the limit of normal, but with persistent complaint of shoulder apprehension, a cognitive-behavioral approach could help them to realize that persistent shoulder apprehension is not necessarily associated with recurrent shoulder instability.

This study has some limitations. The data were analyzed retrospectively, and we cannot exclude some biases. The absence of randomization of patients with or without capsular repair and the higher age of patients without capsular repair are other sources of biases. The heterogeneity of the grous and the low number of patients without capsular repair limit statistical power. A new study with a larger number of patients could enable to meet the initial objective.

In conclusion, half of patients reported persistent shoulder apprehension after Latarjet procedure regardless of capsular repair. Rowe and Walch-Duplay scores had high specificity to identify patients reporting shoulder apprehension. However, their low sensitivity may underestimate persistent shoulder apprehension despite obvious shoulder stability.

## Data Availability

The data that support the findings of this study are available from the corresponding author upon reasonable request.
